# Anterior Cruciate Ligament Tear Using Magnetic Resonance Imaging among Patients Undergoing Arthroscopy in a Tertiary Care Centre: A Descriptive Cross-sectional Study

**DOI:** 10.31729/jnma.7989

**Published:** 2023-05-31

**Authors:** Rohit Shrestha, Sushant Kumar Khadka, Aashutosh Chaudhary, Shreedhar Prasad Acharya, Manasil Malla, Prabesh Gautam, Sagar Maharjan, Ashkal Basi, Sangharsha Thapa, Subindra Karki

**Affiliations:** 1 Department of Orthopedics, Dhulikhal Hospital, Dhulikhel, Kavre, Nepal; 2 Kathmandu University School of Medical Sciences, Dhulikhel, Kavre, Nepal; 3 Kathmandu Medical College and Teaching Hospital, Sinamangal, Kathmandu, Nepal; 4 Department of Radiology, Dhulikhel Hospital, Dhulikhel, Kavre, Nepal

**Keywords:** *anterior cruciate ligament tears*, *arthroscopy*, *cross-sectional studies*, *MRI*

## Abstract

**Introduction:**

Magnetic Resonance Imaging is the preferred imaging modality in patients having anterior cruciate ligament tears. The aim of this study was to find out the prevalence of anterior cruciate ligament tears using magnetic resonance imaging among patients undergoing arthroscopy in a tertiary care centre.

**Methods:**

A descriptive cross-sectional study was conducted in the Department of Orthopaedics and Traumatology of a tertiary care centre. Data from 17 November 2017 to 17 October 2022 were collected between 26 December 2022 and 30 December 2022 from the hospital records. Ethical approval was obtained from Institutinal Review Committee of the same institute (Reference number: 233/22). All patients with a knee injury who received arthroscopy were included in the study. Magnetic resonance imaging reports, arthroscopic findings and relevant data of each case were retrieved from the medical case records of patients. Convenience sampling method was used. Point estimate and 95% Confidence Interval were calculated.

**Results:**

Among patients with arthroscopy confirmed anterior cruciate ligament tear, 138 (91.39%) (86.92 to 95.86, 95% Confidence Interval) had anterior cruciate ligament tear diagnosed with magnetic resonance imaging. The mean age of the patients who had anterior cruciate ligament tear in the magnetic resonance imaging was 32.35±11.31 years. Out of them, 87 (63%) were males and 51 (37%) were females. The mean duration of the injury was 11.60±18.47 months.

**Conclusions:**

The prevalence of anterior cruciate ligament tear using magnetic resonance imaging among patients undergoing arthroscopy in tertiary care centres was similar when compared to other similar studies when conducted in similar settings.

## INTRODUCTION

Anterior cruciate ligament (ACL) is a band of dense connective tissue which can get injured during trauma inducing sudden change in direction of movement, abnormal jumping and landing or direct trauma to the lateral aspect of the knee leading to knee instability.^1^

Magnetic Resonance Imaging is the preferred imaging modality in most patients which can show direct signs of ACL injury.^2^ Whereas, arthroscopy has been traditionally used to diagnose ACL injury.^3^ There are limited studies regarding this modality in our centre.

The aim of this study was to find out the prevalence of anterior cruciate ligament tears using magnetic resonance imaging (MRI) among patients undergoing arthroscopy in a tertiary care centre.

## METHODS

A descriptive cross-sectional study was conducted in the Department of Orthopedics and Traumatology of Dhulikhel Hospital, Dhulikhel, Kavre, Nepal. Data from 17 November 2017 to 17 October 2022 were collected between 26 December 2022 and 30 December 2022 from the hospital records. The ethical approval was taken from the Institutional Review Committee of the same institute (Reference number: 233/22). We included records of all those patients with knee injuries who had arthroscopy performed over the period of the last five years. The decision to perform arthroscopy was made by a team of orthopaedic surgeons. ACL tears when really present in arthroscopy were managed with ACL reconstruction. MRI reports, arthroscopic findings and relevant data of each case were retrieved from medical case records of patients. Patients with incomplete MRI and arthroscopy records and patients who underwent arthroscopy for multi-ligament cases, osteochondral fractures, synovectomy and congenital defects were excluded from the study. The sample size is calculated by using following formula:


$$\begin{array}{ll} \text{n} & ={\text{Z}}^{2}\times \frac{\text{p}\times \text{q}}{{\text{e}}^{\text{2}}}\\ & =1.96^{2}\times \frac{0.955\times 0.045}{0.05^{2}}\\ & =67 \end{array}$$

Where,

n = minimum required sample size
Z = 1.96 at 95% Confidence Interval (CI)
p = prevalence of ACL tear taken from previous study, 9.5%^3^
q = 1-p
e = margin of error, 5%

The calculated sample size was 67. After adding 10% missing data, the sample size was 70. However, 73 sample size was taken.

Data were entered in Microsoft Excel 2016 and analysis was done by using IBM Statistics SPSS 16.0. Point estimate and 95% CI were calculated.

## RESULTS

Among patients undergoing arthroscopy, anterior cruciate ligament tear, 138 (91.39%) (86.92-95.86, 95% CI) had anterior cruciate ligament tear diagnosed with MRI. The mean age of the patients who had ACL tears in the MRI was 32.35±11.31 years. Out of them, 87 (63%) were males and 51 (37%) were females. Among them, 114 (82.6%) were from the hilly district, 18 (13.04%) patients were from Terai and 6 (4.3%) patients were from the Himalayan district. The mean duration of the injury was 11.60±18.47 months. Twisting injuries of the knee while performing activities of daily life were the commonest mode of injury in our study (Table 1).

**Table 1:** Mode of Injury of ACL detected by MRI (n= 138).

Mode of Injury	n (%)
Twisting injury while performing activities of daily life (ADL)	61 (44.20)
Sports injury	33 (23.91)
Fall-related	25 (18.12)
Road traffic accident	16 (11.59)
Can not recall the history of trauma	3 (2.17)

## DISCUSSION

Arthroscopy has been traditionally used to diagnose ACL injury.^3^ Diagnostic accuracy of arthroscopy itself has been reported to be about 95% compared to surgical exploration but it is an invasive procedure and can cause complications like infection, hemarthrosis, adhesions and reflex sympathetic dystrophy.^4,5^ Use of MRI has been increasing as it has been proven as a non-invasive and accurate diagnostic test of ACL injuries which can be subsequently managed by therapeutic arthroscopy, the negating need for diagnostic arthroscopy in the majority of patients.^3,6^

Diagnostic accuracy and sensitivity of MRI in finding ACL tear is found to be 94.85% and 95.45% respectively when comparing against arthroscopy.^3^ But there are many studies with variable results. In a meta-analysis conducted with a 95% CI, the pooled sensitivity (SE), specificity (SP), positive likelihood ratio (LR+), negative likelihood ratio (LR-), and diagnostic odds ratio (DOR) were found to be 87% (84-90%), 90% (88-92%), 6.78 (4.87-9.44), 0.16 (0.13-0.20), and 44.70 (32.34-61.79), respectively.^7^ Among the cases of ACL tear detected by arthroscopy in our study, MRI correctly showed a tear in 138 (91.39%) cases.

MRI carries many advantages as it provides a clear image with high spatial and soft tissue resolution which can facilitate clear observation of knee ligament and meniscus injuries along with evaluation of adjacent structures.^8^ MRI has proven to have high accuracy and good consistency with arthroscopic diagnosis and can reliably guide health care professionals in decision making and can be used as first choice investigation for diagnosis of ACL injury.^3^

In an MRI of ACL injury, the primary sign is fibre discontinuity.^9^ In an acute injury, MRI shows thickening and oedema of ACL characterized by increased signal intensity on T2 or intermediate weighted sequences and in chronic cases, fibres can be completely absorbed or residual ACL stump can show adhesion to the synovial covering of posterior cruciate ligament.^2^

In a complete ACL tear, an empty notch sign can be seen in coronal imaging.^9^

In our study, MRI was detected only in 138 (91.39%) cases. Misdiagnosis of ACL tear by MRI can be seen due to partial tears, haemorrhage and fluid accumulation around ligaments following acute trauma and different scanning angles where the tear may be missed.^3^ Reliance solely on sagittal imaging may lead to incomplete visualization of the contiguous fibres along the complete course of ACL.^10^ Sagittal imaging alone can also lead to errors in the observation of intrasubstance signal intensity.^11^ Thus in addition to sagittal imaging, axial and coronal planes can be taken to allow for complete visualization.^11^

Partial tears are more difficult to diagnose than complete ACL tears in MRI as partial tears can be erroneously reported as mucoid degeneration, complete tears or a normal ACL.^12^ Partial tear may be visualized as fibre laxity or posteroinferior bowing with the increased signal intensity of ACL.^2^ Sagittal imaging alone also makes it difficult to detect partial tears of femoral origin and tears of the isolated bundles alone.^13^ Evaluation of axial images can help in the detection of partial tears of femoral origin and also help to evaluate isolated bundle injuries.^13^ A complete ACL tear may undergo scarring and can get attached to a non-anatomic location.^14^ This may be mistaken for an intact ACL or a partial tear.^15^ In such cases, scarring onto a nonanatomic point and prior imaging if present can help in the diagnosis and assessment of the severity of the original injury.^10^

## CONCLUSIONS

The prevalence of ACL tears using MRI cases among patients undergoing arthroscopy in tertiary care centers was similar when compared to similar studies when conducted in similar settings. MRI can be a sensitive method of diagnosis of ACL tear in comparison to reference standard arthroscopy but misdiagnosis can occur due to partial tear, scarring, haemorrhage and fluid accumulation around ligaments following acute trauma and different scanning angles.
